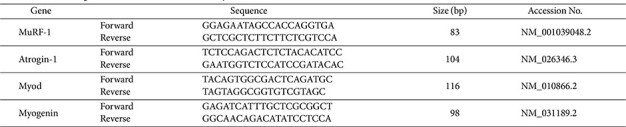# Kimchi Probiotic *Weissella cibaria* Wikim0187 Attenuates Age-Related Muscle Loss: Association with Gut Microbiota Remodeling in Mice

**DOI:** 10.4014/jmb.2607.07010

**Published:** 2026-07-17

**Authors:** Sulhee Lee, Ga Hee Choi, Sang-Pil Choi, Namhee Kim, Young Seo Jang, Min-Sung Kwon, Young Joon Oh, Jeong Hyun Seo, Hwayeon Sun, Ji Ye Mok, Sang Min Park, Byungwook Lee, Hak-Jong Choi

**Affiliations:** 1 Kimchi Healthcare Research Group, World Institute of Kimchi, Gwangju 61755, Republic of Korea; 2 Kimchiome Bio-Resources Research Group, World Institute of Kimchi, Gwangju 61755, Republic of Korea; 3 Korean Culture Center of Microorganisms, Seoul 03641, Republic of Korea; 4 Division of Animal Science, Chonnam National University, Gwangju 61186, Republic of Korea; 5 Division of Radiation Biomedical Research, Korea Institute of Radiological and Medical Sciences, Seoul 01812, Republic of Korea; 6 Department of Biotechnology, Graduate School, Korea University, Seoul 02841, Republic of Korea; 7 Pharmsville Co., Ltd., Seoul 07793, Republic of Korea

**Keywords:** *Weissella cibaria*, Lactic acid bacteria, Kimchi, Sarcopenia, Aging mouse, Muscle function

## Abstract

Sarcopenia, characterized by an age-related progressive decline in skeletal muscle mass and strength, is emerging as a major public health concern in aging societies. Despite growing insights into sarcopenia pathophysiology, effective and targeted therapeutic strategies for preventing or treating age-related muscle loss remain limited. Here, we aimed to examine the effects of *Weissella cibaria* Wikim0187, a probiotic strain isolated from kimchi, on age-related sarcopenia. Male C57BL/6J mice aged 18 months received oral administration of Wikim0187 (1 × 10^9^ CFU per mouse) for 4 months. Wikim0187 supplementation induced significant increases in gastrocnemius muscle mass and quadriceps muscle mass, absolute grip strength, and mean muscle fiber cross-sectional area. At the molecular level, MuRF-1 and Atrogin-1 expression was substantially reduced, whereas that of MyoD and Myogenin was markedly elevated, particularly in the quadriceps muscle. Western blot analysis confirmed a reduction in Atrogin-1 protein levels and an increase in phosphorylated Akt. In addition, gut microbiota analysis revealed significant alterations in microbial composition, with positive correlations between enriched bacterial taxa and improved clinical parameters. Taken together, these findings support the therapeutic potential of Wikim0187 as a safe and effective intervention for the prevention and treatment of sarcopenia in elderly populations.

## Introduction

The progressive loss of muscle mass and function with advancing age not only impairs physical mobility but also increases the risk of falls, fractures, and loss of independence, significantly affecting quality of life in elderly populations [1]. Skeletal muscle is the most abundant and essential tissue in the human body, accounting for approximately 30–40% of total body weight [2]. Muscle mass decreases by 3–8% per decade after age 30, by 1.1–1.4% per year after age 50, and by 2.3% per year after age 60 [3]. Epidemiological studies indicate that approximately 5–10% of the general population is affected by sarcopenia, with prevalence increasing to 10–27% after age 60 [4]. Sarcopenia, characterized by the age-related decline in skeletal muscle mass and strength, has become a major public health concern in aging societies [5]. With the global population aging rapidly, the World Health Organization estimates that by 2030, approximately 1.3 billion individuals worldwide will be aged 60 years or older, underscoring the urgency of developing effective strategies for the prevention and management of sarcopenia [4].

The etiology of age-related muscle loss is multifactorial, involving diverse physiological mechanisms including mitochondrial dysfunction, oxidative stress, chronic inflammation, decreased cellular autophagy, reduced muscle fiber number, malnutrition, alterations in excitation–contraction coupling, hormonal dysregulation, and impaired protein synthesis [6-8]. Among these mechanisms, alterations in gut microbiota composition and diversity contribute substantially to age-related muscle decline, a phenomenon referred to as the gut–muscle axis [9]. Aging-associated dysbiosis has been shown to reduce the production of short-chain fatty acids (SCFAs) and increase intestinal permeability, thereby enhancing systemic inflammation and accelerating muscle wasting [9. 10]. In addition, dysbiotic gut microbiota in older individuals exhibit reduced abundance of beneficial bacteria that produce butyrate and propionate, key metabolites involved in maintaining intestinal barrier integrity and supporting immune homeostasis [11].

Oxidative stress represents a critical contributor to sarcopenia. An imbalance between reactive oxygen species (ROS) production and antioxidant defence during aging leads to cellular damage and impaired muscle regeneration [12]. Skeletal muscle, which contains a high density of mitochondria, is particularly susceptible to oxidative damage. This oxidative burden activates catabolic signaling pathways, particularly the ubiquitin–proteasome system (UPS), resulting in the upregulation of muscle-specific E3 ubiquitin ligases such as MuRF-1 (muscle RING-finger protein-1) and Atrogin-1, thereby accelerating muscle protein degradation [13, 14]. Activation of Forkhead box O (FoxO) transcription factors, driven by oxidative stress and inflammatory signaling, further promotes the expression of atrophy-related genes and sustains the catabolic state [15, 16]. The molecular mechanisms underlying age-related sarcopenia remain incompletely understood, and no pharmacological therapies have yet been approved for its treatment [17]. The development of effective interventions remains an urgent priority for promoting healthy aging.

Protein intake, which is essential for muscle synthesis, is often limited in the elderly due to anabolic resistance caused by aging [18]. This highlights the need for new nutritional strategies to maintain muscle homeostasis. Consequently, regulating the gut environment through probiotics is emerging as an approach that supports the gut-muscle axis. Probiotics, defined as live microorganisms that confer health benefits to the host, have emerged as promising therapeutic candidates for managing age-related disorders. Lactic acid bacteria (LAB), particularly species belonging to the genus *Weissella*, have been extensively studied for their immunomodulatory, anti-inflammatory, and antioxidant properties [19-22]. These beneficial microorganisms can modulate gut microbiota composition, enhance intestinal barrier function, and promote the production of bioactive metabolites that support muscle health [23-25]. The protective effects of LAB are mediated through multiple mechanisms, including direct antioxidant activity via the production of reactive oxygen species–scavenging metabolites, modulation of intestinal dendritic cell populations, and biosynthesis of secondary metabolites such as folate and B vitamins that support cellular energy metabolism [26, 27].

Epidemiological studies have associated kimchi consumption with various health benefits, including improved metabolic profiles and reduced risk of age-related diseases [28, 29]. *Weissella cibaria* is a non-pathogenic, Gram-positive lactic acid bacterium commonly isolated from traditional fermented foods, particularly kimchi, a staple Korean food with a long history of safe consumption [30, 31]. Kimchi, produced through the fermentation of vegetables such as cabbage, radish, red pepper, and garlic, undergoes microbial transformation primarily mediated by LAB, including *Leuconostoc*, *Lactobacillus*, and *Weissella* species [32]. *Weissella* species are dominant species found in large quantities during the mid-stage of kimchi fermentation and are recognized as potential probiotics due to their acid tolerance and ability to adhere to the intestinal epithelium [33, 34]. Although *Weissella* species have been investigated for their beneficial effects in inflammatory bowel disease, immune disorders, atopy, and obesity [20, 35, 36], their potential role in mitigating sarcopenia has not been comprehensively examined in aging models.

Therefore, the primary objective of this study was to comprehensively evaluate the protective efficacy and underlying molecular mechanisms of the kimchi-derived probiotic *Weissella cibaria* Wikim0187 against age-related sarcopenia. By utilizing both *in vitro* myoblast models and an *in vivo* naturally aged mouse model, we aimed to determine whether Wikim0187 could effectively attenuate muscle wasting by modulating oxidative stress, protein turnover pathways, and the gut microbiota.

## Experimental

### Preparation of *W. cibaria* Wikim0187

*W. cibaria* Wikim0187 was isolated from cabbage kimchi, a traditional Korean fermented food, and deposited in the Korean Federation of Culture Collection under the accession number KFCC11987P. The strain was routinely cultured in Lactobacilli MRS broth (de Man, Rogosa, and Sharpe broth; BD Difco, USA) at 30°C with an overnight incubation. The viable cell count was determined by measuring the absorbance at 600 nm using a spectrophotometer, and the culture was then used for examination in muscle cell lines in *in vitro* experiments. *W. cibaria* Wikim0187 was cultured to full growth, suspended in 25% (v/v) glycerol, and stored at -70°C until use to ensure strain consistency. The freeze-dried *W. cibaria* Wikim0187 was prepared by Pharmsville Co., Ltd., and an appropriate amount of the freeze-dried culture was reconstituted in PBS prior to oral gavage.

### *In Vitro* Cell Culture and Experiments

C2C12 myoblasts (murine skeletal muscle–derived cells; ATCC CRL-1772, USA) were cultured in Dulbecco’s modified Eagle’s medium (DMEM; HyClone, USA) supplemented with 10% fetal bovine serum (FBS; HyClone), 1% penicillin–streptomycin (P/S; HyClone), and 2 mM L-glutamine at 37°C in a humidified atmosphere containing 5% CO_2_.

For myoblast viability and protection assays, C2C12 myoblasts harvested at 80–85% confluence were seeded at a density of 1 × 10^5^ cells/well in 24-well plates and cultured for 24 h to allow attachment. Subsequently, the cells were treated with various concentrations of live *W. cibaria* Wikim0187 diluted in serum-free DMEM for 24 h. For the hydrogen peroxide (H_2_O_2_)-induced cytotoxicity model, cells were pretreated with Wikim0187 at specified concentrations for 24 h, followed by exposure to 1 mM H_2_O_2_ for an additional 4 h. Cell viability was assessed using the CCK-8 assay kit (Enzo Life Science, USA). Results were normalized to those of the vehicle control group and expressed as percentages of viable cells. Creatine kinase (CK) activity in the culture medium was measured as an indicator of muscle cell membrane integrity and cellular damage. Following treatment with various concentrations of Wikim0187, culture supernatants were collected and centrifuged at 1,000 × g for 5 min to remove cellular debris. CK activity was quantified using a commercial creatine kinase assay kit (ab155901, Abcam, UK) according to the manufacturer’s instructions.

### Animal Experiments

Male C57BL/6J mice aged 8 weeks at the start of the study were purchased from OrientBio (Republic of Korea) and aged to 18 months, a stage corresponding to an aged mouse model, which is well-characterized as exhibiting age-related sarcopenia comparable to that observed in humans aged 56–60 years based on comparative lifespan analysis. Animals were housed individually in polypropylene cages under controlled environmental conditions (temperature: 22 ± 2°C; humidity: 50–60%; 12 h light/dark cycle with lights on at 06:00 and off at 18:00). Mice had ad libitum access to a standard laboratory diet (Purina, Republic of Korea) containing 22% protein and 5% fat, as well as autoclaved water. All animals were acclimated to the housing conditions for seven days prior to experimental procedures and were monitored daily for signs of illness or distress. All experimental procedures were approved by the Institutional Animal Care and Use Committee (IACUC) of the World Institute of Kimchi Research (approval number: WIKIM IACUC 202238) and were conducted in accordance with the National Institutes of Health Guide for the Care and Use of Laboratory Animals. Aged mice were randomly assigned to two groups: a vehicle group (n = 10) receiving sterile PBS and a W0187 group (n = 10) receiving *W. cibaria* Wikim0187. The freeze-dried product administered to the W0187 group was suspended in PBS at a concentration of 1 × 10^9^ colony-forming units (CFU) per mouse in a total volume of 200 μL and administered orally once daily for 4 months (16 weeks). This dose was selected based on preliminary dose–response experiments and represents a physiologically relevant probiotic intake. The vehicle group received an equivalent volume and weight of freeze-dried material without bacterial culture, suspended in PBS, for the same duration. Body weight was recorded weekly using a calibrated electronic balance, and all behavioral and functional assessments were performed at regular intervals throughout the treatment period. The groups and schemes of experimental animals are shown in Fig. 2A.

### Dual-Energy X-ray Absorptiometry (DXA) Analysis

Whole-body composition, including fat mass, lean mass, and bone mineral density (BMD), was assessed using a small-animal DXA scanner (iNSiGHT VET DXA, OsteoSys, Republic of Korea, or Hologic Discovery A, USA) at baseline and after 4, 8, 12, and 16 weeks of treatment. Mice were anesthetized with isoflurane inhalation in a chamber (Hana Pharm Co., Ltd., Republic of Korea), prior to scanning. X-ray images and color-coded distribution maps were generated to visualize regional bone density. All scans were performed by an operator blinded to group allocation.

### Muscle Mass Measurement

At the end of the 4-month (16-week) treatment period, mice were sacrificed under CO_2_ asphyxiation conditions. The gastrocnemius (GA), tibialis anterior (TA), and quadriceps (QR) muscles from both hindlimbs were rapidly excised, carefully cleared of connective and adipose tissues, blotted to remove excess moisture, and weighed using a precision balance (Mettler-Toledo, USA) with a resolution of 0.1 mg. All muscle samples were collected and weighed within 30 min of sacrifice to minimize postmortem changes.

### Assessment of Muscle Strength and Function

Forelimb Grip Strength Test: Forelimb grip strength was measured using a digital grip strength meter (Ugo Basile SRL, Italy) at monthly intervals throughout the 4-month treatment period. Mice were gently suspended by the tail and allowed to grasp a metal grid or bar connected to the force transducer with their forelimbs. Five to fifteen consecutive measurements were recorded for each mouse at each time point, and the maximum grip force (in grams) was used for analysis after excluding the highest and lowest measurements. To account for inter-individual differences in body weight, grip strength values were normalized to body weight and expressed as grip strength per gram of body weight. All measurements were conducted between 09:00 and 12:00 h by an investigator blinded to group allocation.

Rotarod Performance Test: Motor coordination and muscle endurance were assessed using an accelerating rotarod apparatus equipped with a 7-cm diameter rotating rod or a five-lane Rota-Rod system (Ugo Basile SRL). Mice were placed on the rotating rod, which accelerated from 4 to 40 rpm over a 5-min period (300 s). The latency to fall, defined as the time each mouse remained on the rotating rod, was recorded as a measure of balance, coordination, and muscular endurance. Each mouse underwent three trials per session with a 30-min inter-trial interval to allow recovery. Testing was conducted monthly throughout the 4-month experimental period, with a maximum cutoff time of 5 min per trial.

### Histological Analysis and Muscle Fiber Measurements

Muscle tissue samples (GA and TA) were rapidly excised, embedded in optimal cutting temperature (OCT) compound or fixed in 10% formalin (Sigma, USA), and either frozen in liquid nitrogen or embedded in paraffin at −80°C for preservation. Serial cross-sections (5–10 μm thickness) were prepared using a cryostat (Leica CM3050S, Germany) set at −20°C or using a microtome, and mounted onto pre-coated glass slides. For general histological evaluation, sections were stained with hematoxylin and eosin (H&E) using standard protocols to assess muscle fiber morphology and the presence of inflammatory infiltrates. Muscle fiber cross-sectional area (CSA) and minimum Feret diameter (MFD) were quantitatively analyzed using image analysis software (ImageJ, National Institutes of Health, USA) at 400× and 50× magnification. At least 200–300 muscle fibers were analyzed per animal for each muscle group, with fibers selected from both central and peripheral regions of the muscle cross-section.

### Gene Expression Analysis by Quantitative Real-time PCR

Total RNA was extracted from frozen muscle tissues (GA, TA, and QR) using TRIzol reagent (Invitrogen, USA) according to the manufacturer’s protocol. Tissue samples were homogenized in TRIzol using a PowerGen 125 tissue homogenizer or standard methods. First-strand cDNA was synthesized from total RNA using a reverse transcription system (SuperScript II, Invitrogen, or TOPscript™ cDNA Synthesis Kit, Enzynomics, Republic of Korea) with oligo(dT) primers. Quantitative real-time PCR (qRT-PCR) was performed using a StepOnePlus Real-Time PCR System (Applied Biosystems, USA) or Bio-Rad CFX96 system (Bio-Rad, USA) with SYBR Green-based detection. Gene expression levels were quantified using the comparative Ct (^ΔΔCt^) method with normalization to the housekeeping gene GAPDH. The primers used for qRT-PCR are listed in Table 1.

### Western Blot Analysis

Muscle tissue samples (left TA muscle) were rapidly excised and immediately lysed in radioimmunoprecipitation assay (RIPA) buffer (50 mM Tris-HCl, pH 7.4; 150 mM NaCl; 1% Triton X-100; 1% sodium deoxycholate; 0.1% SDS) supplemented with protease and phosphatase inhibitor cocktails (Roche Diagnostics, Germany). Lysates were incubated on ice for 30 vortexed every 10 min, and centrifuged at 12,000 × g for 15 min at 4°C. The soluble protein fraction was collected and stored at −80°C until analysis. Total protein concentration was determined using the Bradford assay with bovine serum albumin (BSA) as the standard. Protein samples were derived from TA muscles and electrophoretically separated by SDS-PAGE and transferred onto polyvinylidene fluoride membranes (Millipore, USA). Membranes were blocked with 5% non-fat milk or 5% (w/v) BSA in Tris Buffered Saline (TBS) and then incubated with primary antibodies, including anti-Atrogin-1 (sc166806, Santa Cruz Biotechnology, USA), anti-phosphorylated Akt (p-Akt, Ser473, 9271s, Cell Signaling Technology, USA), anti-total Akt (9272s, Cell Signaling Technology), and anti-GAPDH (ab9484, Abcam, UK). After washing, membranes were incubated with horseradish peroxidase (HRP)-conjugated secondary antibodies (7074, Cell Signaling Technology) for 1 hour at room temperature. Protein bands were visualized ECL detection kit (Bio-Rad) and analyzed with a chemiluminescent imager (Amersham Biosciences, USA) and quantified by densitometric analysis using ImageJ software. Data were normalized to GAPDH as a loading control.

### Gut Microbiota Analysis

Cecal contents were collected and stored at −80°C until analysis. Cecal microbiota profiling was performed by EZBioCloud using 16S rRNA gene amplicon sequencing targeting the V3–V4 regions on an Illumina MiSeq platform, yielding an average of 31,064 reads per sample. Low-quality reads were filtered out based on the following criteria: sequence length <100 bp or >2,000 bp, average Q-score <25, and failure to be predicted as a 16S gene by Hidden Markov Model-based search; putative singleton sequences were also excluded. β-diversity was assessed using Bray–Curtis distances, and principal coordinates analysis (PCoA) was employed to visualize differences in community structure between the Vehicle and *W. cibaria* Wikim0187 groups. Differentially abundant taxa between groups were identified using linear discriminant analysis effect size (LEfSe) analysis, with only taxa exceeding the predefined LDA threshold and significance level being retained. Correlations between the relative abundances of key taxa and sarcopenia-related parameters were evaluated using Pearson’s correlation test, and the results were summarized in a correlation heatmap. Relative abundances of selected taxa were compared between groups and are presented as mean ± SD, with statistical significance set at *p* < 0.05.

### Statistical Analysis

All data are presented as mean ± standard error of the mean (SEM). Statistical comparisons between the vehicle and Wikim0187-treated groups were performed using unpaired Student’s *t*-tests for two-group comparisons or one-way analysis of variance (ANOVA) followed by Šídák's or two-tailed post-hoc multiple comparison tests for comparisons among multiple groups. For repeated measurements over time, a mixed-effects model was used to accommodate missing data, followed by Šídák's multiple comparison test to evaluate the interaction between time and treatment. Statistical analyses were conducted using GraphPad Prism version 9.0 or later (GraphPad Software, USA). Significance levels were set at *p < 0.05, **p < 0.01, ***p < 0.001, and ****p < 0.0001.

## Results

### *In Vitro* Cytoprotective Effects of *W. cibaria* Wikim0187 on C2C12 Myoblasts

**Fig. 1:**
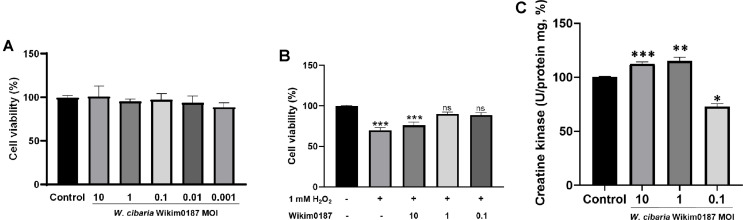
Effects of *W. cibaria* Wikim0187 on growth of C2C12 myoblasts and creatine kinase activity. (**A**) Viability of C2C12 cells treated with Wikim0187. (**B**) Protective effects of Wikim0187 against H_2_O_2_-induced cytotoxicity in C2C12 cells. (**C**) Creatine kinase activity in C2C12 cells treated with various concentrations of Wikim0187. Data are presented as the mean ± SEM (n = 3–4). Statistical analysis was performed using one-way ANOVA (*p < 0.05, **p < 0.01, or ***p < 0.001 vs. control).

To evaluate the protective effects of *W. cibaria* Wikim0187 on muscle cells, *in vitro* experiments were conducted using C2C12 myoblasts. Treatment with Wikim0187 did not significantly affect the basal viability of C2C12 cells across the tested concentration range (MOI 0–10), indicating that the strain is non-toxic to muscle cells at the concentrations examined (Fig. 1A). However, when cells were exposed to oxidative stress induced by 1 mM H_2_O_2_, pretreatment with Wikim0187 conferred significant cytoprotective effects. H_2_O_2_-induced cytotoxicity was markedly reduced in cells pretreated with Wikim0187, with maximal protection observed at MOI values of 1 and 10, demonstrating a concentration-dependent protective effect against oxidative stress–induced cellular damage (Fig. 1B). Treatment with Wikim0187 resulted in a dose-dependent reduction in CK activity in the culture medium compared with the control group, with statistically significant reductions observed at higher concentrations (MOI of 1 and 10, *p* < 0.05). Specifically, treatment at an MOI of 0.1 reduced CK activity by approximately 27% relative to the control group (Fig. 1C). These findings indicate that Wikim0187 enhances muscle cell membrane integrity and reduces cellular damage, thereby protecting muscle cells from oxidative stress and muscle fatigue, supporting its potential therapeutic use for muscle protection.

### Effects of *W. cibaria* Wikim0187 on Body Weight and Body Composition in Aged Mice

**Fig. 2:**
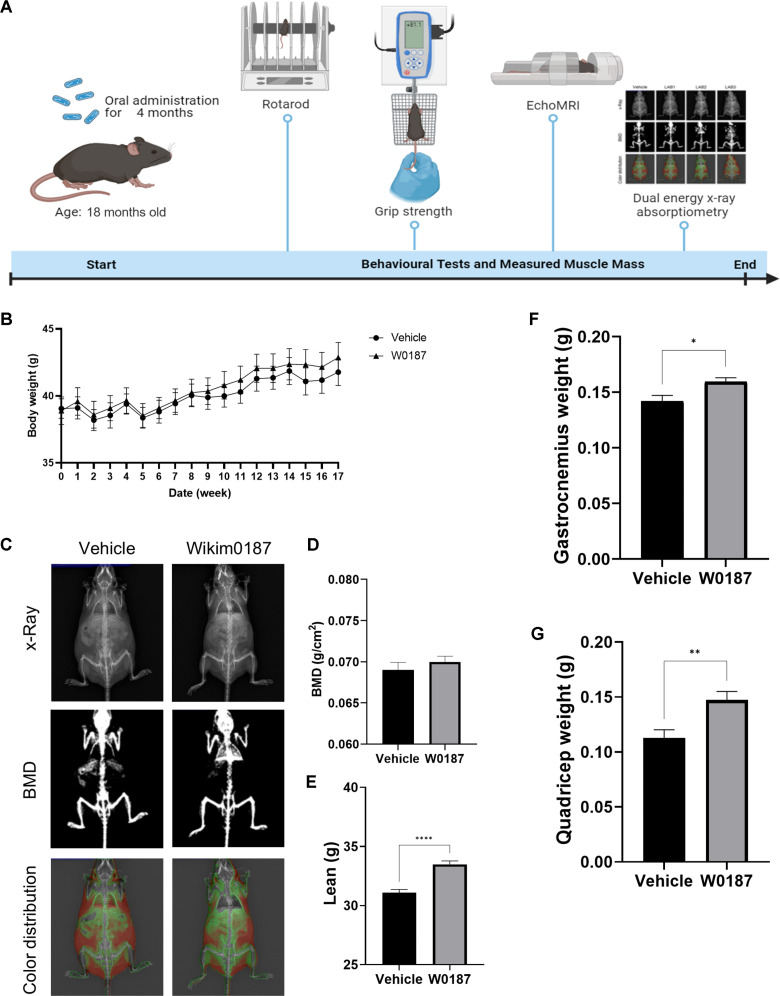
Effects of *W. cibaria* Wikim0187 on body weight and muscle mass in aged mice. (**A**) Experimental scheme. Mice received Wikim0187 (1 × 10^9^ CFU/mouse) daily for 4 months. (**B**) Body weight of vehicle- and Wikim0187-treated groups. (**C**) Whole-body composition images. (**D**) Bone mineral density (BMD) and (**E**) lean mass measured using a DXA scanner. (**F**) Gastrocnemius (GA) and (g) quadriceps (QR) muscle weights. All data are expressed as mean ± SEM (n = 10 per group). Statistical analysis was performed using a *t*-test (*p < 0.05, **p < 0.01, or ****p < 0.0001 vs. vehicle group).

To examine the effect of *W. cibaria* Wikim0187 on sarcopenia in aged mice, *W. cibaria* Wikim0187 was administered orally to 18-month-old mice daily for 4 months (Fig. 2a). Over the 4-month treatment period, vehicle-treated aged mice exhibited progressive age-related changes in body composition. Although body weight in the W0187-treated group increased more slowly than in the vehicle group, the difference did not reach statistical significance (Fig. 2B). However, whole-body composition analysis using DXA revealed significant differences between the two groups (Fig. 2C). Bone mineral density (BMD) and lean mass measurements demonstrated that W0187 treatment significantly preserved lean body mass and maintained BMD compared with the vehicle group (Fig. 2D). The lean mass of the W0187-treated group was significantly higher by 7.7% relative to that in the vehicle group (Fig. 2E). In the color-coded DXA scans, in which lean tissue is displayed in green and fat tissue in red, mice in the W0187 group exhibited a visibly greater proportion of lean tissue compared with vehicle-treated controls (Fig. 2C). Detailed analysis of individual muscle weights further demonstrated that Wikim0187 supplementation significantly increased muscle mass in multiple hindlimb muscles. Gastrocnemius (GA) muscle weight was significantly higher in the W0187-treated group than in the vehicle group, representing a 12.4% increase (Fig. 2F). Quadriceps (QR) muscle weight was also significantly elevated following Wikim0187 treatment, with a 30.4% increase compared with the vehicle group (Fig. 2G). These findings indicate that oral administration of Wikim0187 effectively counteracts age-related skeletal muscle loss across multiple muscle groups, representing a clinically significant protective effect against sarcopenia.

### Effects of *W. cibaria* Wikim0187 on Muscle Strength and Function

**Fig. 3:**
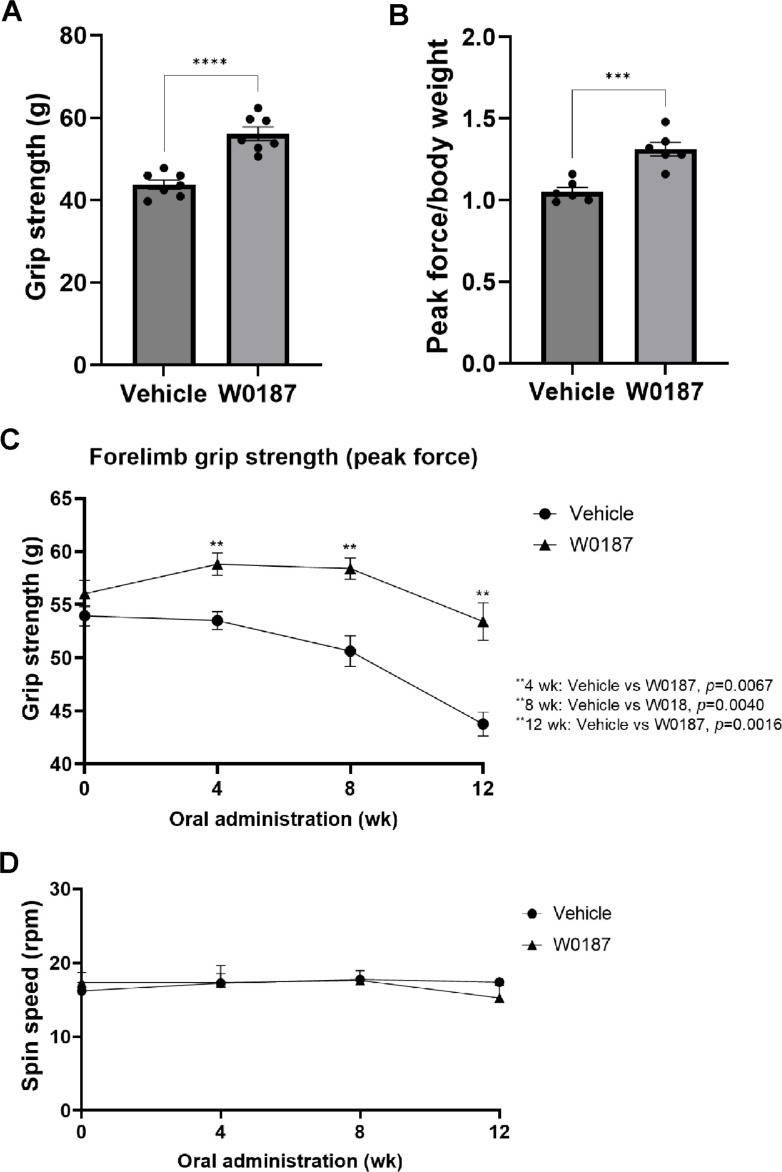
Assessment of the effects of *W. cibaria* Wikim0187 on muscle strength and function in aged mice. (**A**) Forelimb grip strength and (**B**) grip strength normalized to body weight. Statistical analysis was performed using a t-test (***p < 0.001 and ****p < 0.0001). (**C**) Peak forelimb grip force measured monthly. (**D**) Time to fall from an accelerating rotarod. Data presented in panels (**C**) and (**D**) were analyzed using a mixed-effects model followed by Šídák's multiple comparison test to evaluate the interaction between time and treatment (**p < 0.01 vs. vehicle group).

Forelimb grip strength, a well-established indicator of muscle strength and functional capacity, was significantly improved in aged mice treated with Wikim0187 compared to that in the vehicle-treated controls (Fig. 3A). Absolute grip strength showed robust improvement, with values markedly higher in the W0187 group, showing a 28% increase relative to those in the controls. When normalized to body weight, grip strength per unit body weight remained significantly elevated in the W0187-treated group, showing increases of 24%, indicating improved muscle quality and functional capacity independent of body mass differences (Fig. 3A and 3B). Monthly monitoring of forelimb grip strength throughout the treatment period revealed progressive improvements in the W0187-treated group. After weeks of treatment, grip strength was already significantly improved in the W0187 group, with further enhancements observed at 8 weeks and 12 weeks, indicating sustained and progressive beneficial effects of Wikim0187 supplementation on muscle strength development over the treatment period (Fig. 3C). Both groups showed a gradual decline in grip strength over time, consistent with age-related decline in muscle function; however, the W0187-treated group maintained significantly higher grip strength than the Vehicle group, and the decline in strength occurred more slowly, suggesting that Wikim0187 slowed the rate of age-related muscle strength loss. Motor coordination and endurance were assessed using the accelerating rotarod test. W0187-treated mice exhibited longer latency to fall compared with vehicle-treated controls, although the differences did not reach statistical significance at all time points (Fig. 3D). These functional improvements parallel the observed improvements in muscle mass and strength and suggest that Wikim0187 contributes to the preservation of overall muscle physiology in aged mice. A continuous decline in motor function was observed in both groups as aging progressed, consistent with the inherent motor decline associated with aging.

### Effects of *W. cibaria* Wikim0187 on Muscle Fiber Morphology and Cross-Sectional Area

**Fig. 4:**
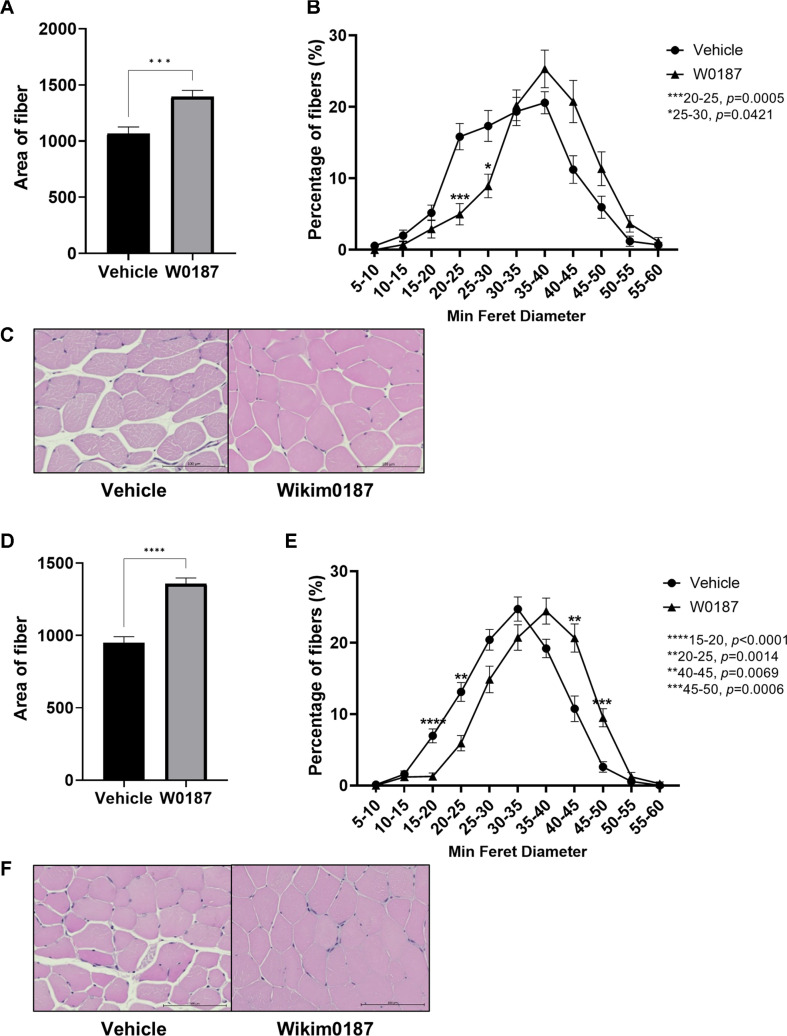
Fiber cross-sectional area (CSA) and minimum Feret diameter of muscle tissue in aged mice. (**A**) Quantitative analysis of fiber CSA of gastrocnemius (GA) muscle. (**B**) Percentage distribution and mean minimum Feret diameter in the GA muscle. (**C**) H&E staining of GA tissue. (**D**) Quantitative analysis of fiber CSA of tibialis anterior (TA) muscle. (**E**) Percentage distribution and mean minimum Feret diameter in TA muscle. (**F**) H&E staining of TA tissue. Data are expressed as mean ± SEM (n = 10). Statistical analysis for (**A**) and (**D**) was performed using a *t*-test (***p < 0.001, ****p < 0.0001). Data for (**B**) and (**E**) were analyzed using one-way ANOVA followed by Šídák’s multiple comparison test (*p < 0.05, **p < 0.01, ***p < 0.001, and ****p < 0.0001 vs. vehicle group).

We investigated whether *W. cibaria* Wikim0187 affects muscle fiber cross-sectional area (CSA) by measuring the muscle fiber CSA of a ged mice. Quantitative analysis of muscle fiber CSA demonstrated that Wikim0187 treatment significantly increased mean fiber CSA in both the GA muscle (31.0% increase) and TA muscle (42.8% increase), indicating substantial enhancement of muscle fiber size (Fig. 4A and 4D). Analysis of minimum Feret diameter (MFD) distributions revealed distinct shifts in fiber size composition. In the GA muscle, the MFD distribution showed a higher proportion of fibers in the 35–40 μm range in the W0187-treated group (Fig. 4B). In the TA muscle, the MFD distribution demonstrated even more pronounced changes, with significantly greater proportions of fibers in the 40–45 μm and 45–50 μm ranges in the W0187-treated group compared with the Vehicle group (Fig. 4E). In H&E-stained sections of TA, the spacing between muscle fibers appeared tighter, and the inter-fiber spaces were visibly reduced in the W0187-treated group compared with the Vehicle group, indicating increased muscle fiber packing density (Fig. 4F). These findings indicate a shift toward larger, more mature muscle fibers, consistent with improved muscle quality and strength.

### Gene Expression of Muscle Atrophy and Growth-Related Markers by *W. cibaria* Wikim0187

**Fig. 5:**
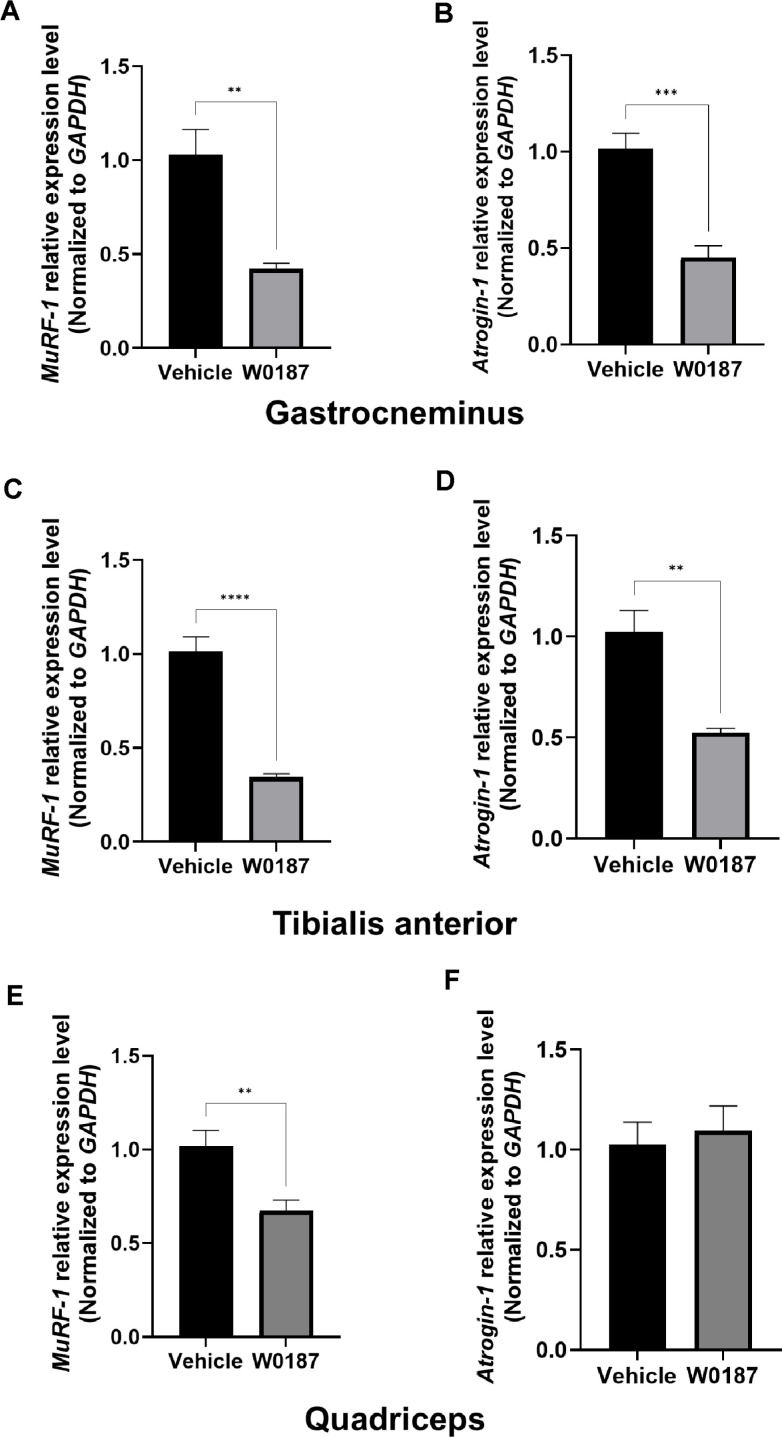
Expression of muscle atrophy-related genes (MuRF-1 and Atrogin-1) in hindlimb muscle tissues. Gene expression levels of (**A**) MuRF-1 in GA tissue, (**B**) Atrogoin-1 in GA tissue, (**C**) MuRF-1 in TA tissue, (**D**) Atrogoin-1 in TA tissue, (**E**) MuRF-1 in QR, and (**F**) Atrogoin-1 in QR. Data are expressed as the mean ± SEM. Statistical analysis was performed using a *t*-test (**p < 0.01, ***p < 0.001, and ****p < 0.0001). (MuRF-1, Muscle RING-finger protein 1; W0187, *W. cibaria* Wikim0187.)

**Fig. 6:**
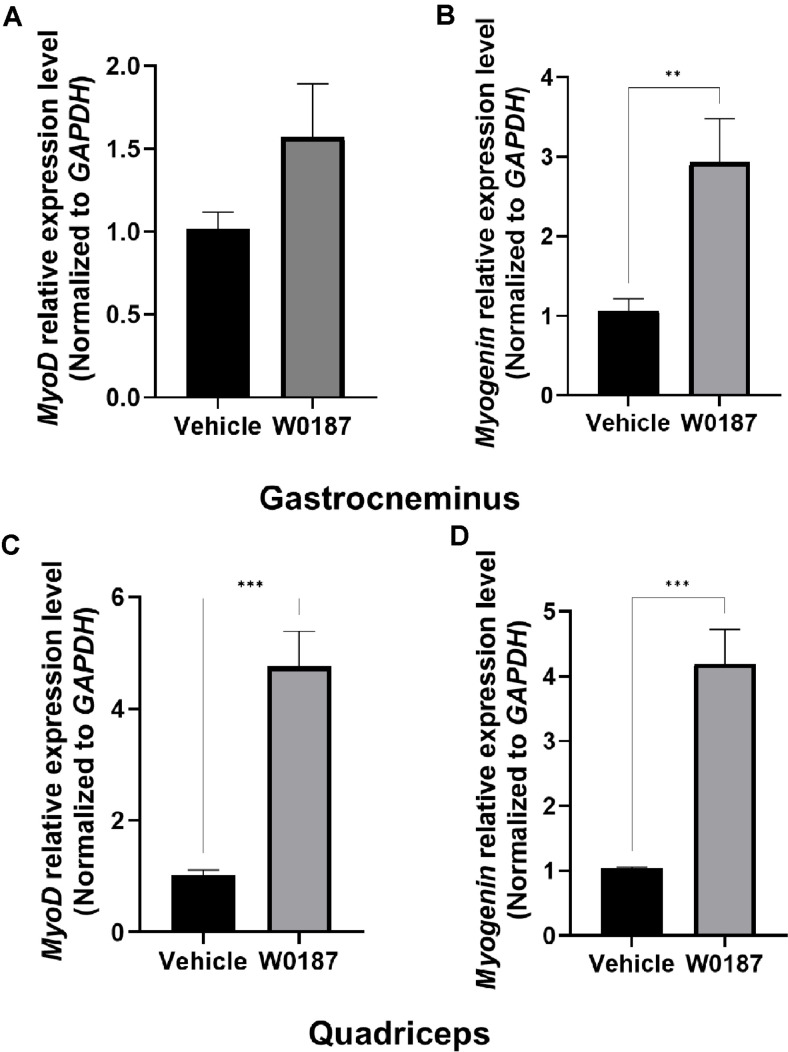
Expression of muscle growth-related genes (MyoD and Myogenin) in hindlimb muscle tissues. Gene expression levels of (**A**) MyoD in GA tissue, (**B**) Myogenin in GA tissue, (**C**) MyoD in QR tissue, and (**D**) Myogenin in QR tissue. Data are expressed as the mean ± SEM. Statistical analysis was performed using a *t*-test (**p < 0.01 and ***p < 0.001).

To further elucidate the mechanism through which *W. cibaria* Wikim0187 intervention improves age-related sarcopenia, we analyzed gene expression related to muscle synthesis and degradation using qPCR. Gene expression analysis demonstrated that Wikim0187 treatment significantly suppressed the expression of muscle atrophy–related genes across multiple hindlimb muscles. MuRF-1 expression was substantially reduced in the W0187-treated group compared with vehicle controls, with reductions of 59.0% in GA muscle (Fig. 5A), 66.1% in TA muscle (Fig. 5C), and 33.9% in QR muscle (Fig. 5E). Similarly, Atrogin-1 expression was significantly decreased following Wikim0187 treatment, with reductions of 55.6% in the GA muscle (Fig. 5B) and 48.8% in the TA muscle (Fig. 5D), whereas a downward trend was also observed in the QR muscle, although the reduction did not reach statistical significance (Fig. 5F). The remarkable reduction in these muscle-specific E3 ubiquitin ligases indicates that Wikim0187 suppresses the ubiquitin-proteasome system-mediated protein degradation pathway, thereby reducing muscle protein catabolism and preventing age-related muscle loss [36, 37]. To assess effects on muscle growth and regeneration, the expression of myogenic regulatory factors MyoD and Myogenin was examined. In the GA muscle, both MyoD and Myogenin expression levels were significantly elevated in the W0187-treated group compared to vehicle controls, with Myogenin increased by 174.8% (Fig. 6A and 6C). In the QR muscle, expression levels of MyoD and Myogenin were significantly elevated, with MyoD showing 367.2% and Myogenin 306.6% increase compared to those in the Vehicle group (Fig. 6B and 6D). These elevated levels of myogenic regulatory factors indicate enhanced myogenic capacity and regenerative potential in aged muscles treated with Wikim0187, suggesting enhanced myoblast proliferation, differentiation, and fusion into functional myotubes, thereby contributing to the increased muscle fiber size and improved functional capacity.

### Mechanisms of Muscle Protein Regulation by *W. cibaria* Wikim0187

**Fig. 7:**
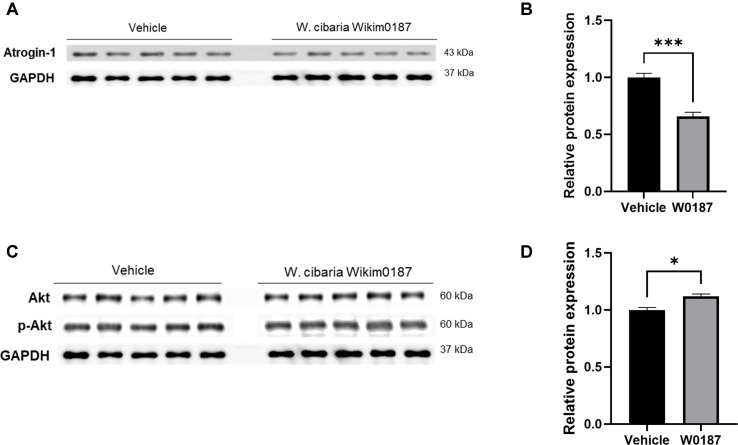
Protein expression levels of muscle markers in TA. (**A**) Representative western blot image of Atrogin-1 protein extracted from TA tissue. Atrogin-1 is observed at approximately 43 kDa. (**B**) Quantification of Atrogin-1 protein expression relative to GAPDH, shown as fold change versus the vehicle group. (**C**) Representative western blot images of total Akt and phosphorylated Akt (p-Akt). Both bands are observed at approximately 60 kDa. Data are expressed as mean ± SEM. Statistical analysis was performed using a *t*-test (*p < 0.05).

To determine the mechanism of *W. cibaria* Wikim0187 in inhibiting sarcopenia induced by aging, phosphorylation levels of Atrogin-1, a protein that regulates degradation, and Akt (protein kinase B), which promotes muscle growth and synthesis, were investigated in TA tissues of aged mice. Atrogin-1 protein was identified with a distinct band at approximately 43 kDa and quantified by normalization with GAPDH (Fig. 7A). Its expression was significantly reduced in the W0187-treated group compared to that in the vehicle controls (34% reduction) (Fig. 7B). Analysis of the phosphatidylinositol 3-kinase/protein kinase B (PI3K/Akt) signaling pathway, a critical regulator of protein synthesis and cell survival, revealed increased activation of Akt in response to Wikim0187 treatment. Akt and phosphorylated Akt (p-Akt), detected at approximately 60 kDa, were significantly elevated in the W0187-treated group (12% increase) compared to those in vehicle controls (Fig. 7C and 7D), indicating activation of anabolic signaling pathways that promote muscle protein synthesis and inhibit catabolic processes. These findings provide mechanistic support for the observed improvements in muscle mass and function, as Akt plays a critical role in both muscle protein synthesis and degradation through multiple downstream signaling pathways.

### Gut Microbiota Modulation by *W. cibaria* Wikim0187

**Fig. 8:**
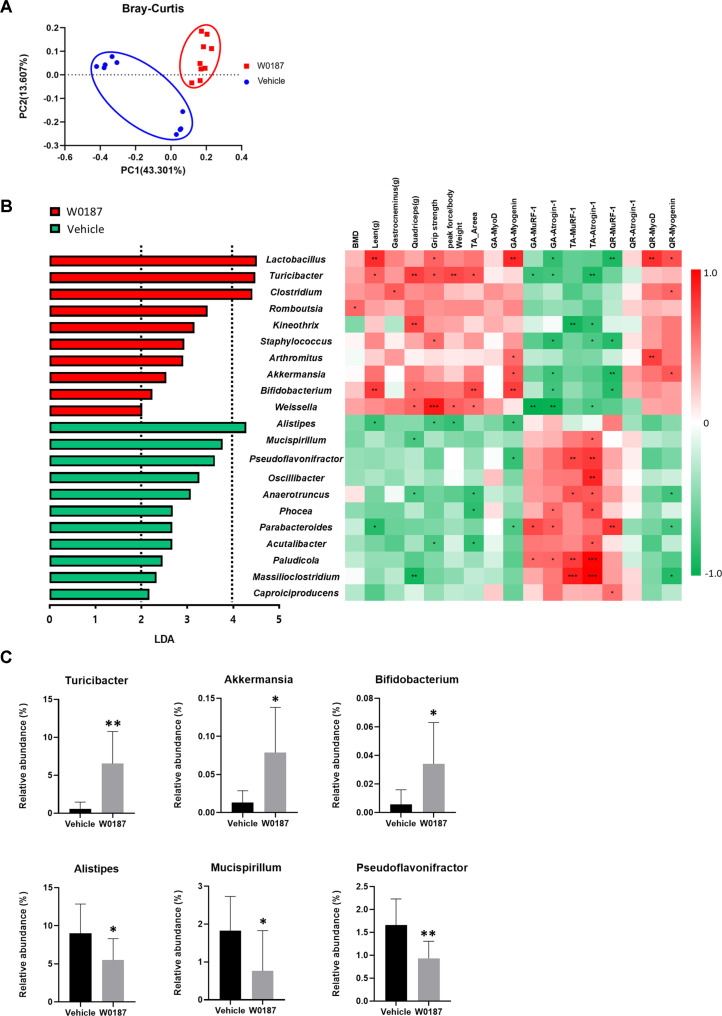
Differentially represented species between the Vehicle and *W. cibaria* Wikim0187 group through linear discriminant analysis effect size (LEfSe) analysis. (**A**) β-diversity analysis comparing Vehicle and W0187 groups. (**B**) Linear discriminant analysis (LDA) scores indicate effect sizes. Correlations between gut microbiota composition and clinical parameters related to obesity are shown as a heat map. Pearson correlation values were used for the matrix, with red indicating positive correlations and blue indicating negative correlations. (**C**) Relative abundance of cecal genus-level microbiota in the Vehicle and W0187 group. Values are presented as mean ± SD (n = 7 per group). *p < 0.05, **p < 0.01, ***p < 0.001 vs. Vehicle group.

To investigate the effect of *W. cibaria* Wikim0187 administration on changes in the gut microbiota community in sarcopenia in aged mice, a gut microbiome study was conducted. β-diversity analysis showed clear separation between the Vehicle and W0187-treated groups, indicating distinct microbial community structures (Fig. 8A). LEfSe analysis with LDA score threshold ≥ 2.0 revealed distinct patterns of microbiota compositional change between the Vehicle control and Wikim0187 groups. Administration of *W. cibaria* Wikim0187 resulted in significant enrichment of bacterial genera such as *Turicibacter*, *Romboutsia*, *Arthromitus*, *Akkermansia*, *Bifidobacterium* (Fig. 8B). Conversely, the Vehicle group demonstrated enrichment of *Alistipes*, *Mucispirillum*, Oscillibacter, *Pseudoflavonifractor*, indicating reciprocal compositional shifts by Wikim0187 supplementation. The genera exhibiting the highest LDA scores in the Wikim0187 group (*Turicibacter*, *Akkermansia*, and *Bifidobacterium*) also showed a proportional increase in relative abundance (Fig. 8C). Notably, the genera enriched by Wikim0187 administration exhibited significant correlations with markers of skeletal muscle integrity and function. The genera *Turicibacter*, *Akkermansia*, and *Bifidobacterium*, which were increased by Wikim0187, displayed positive associations with muscle mass, grip strength, and anabolic myogenic gene expression markers, including GA-Myogenin, QR-MyoD, and QR-Myogenin (Fig. 8B). In contrast, these same enriched genera displayed significant negative correlations with catabolic muscle atrophy-related genes, namely GA-Atrogin1, TA-Atrogin1, and GA-MuRF-1 (Fig. 8B). These microbiota-phenotype correlations suggest that Wikim0187-induced microbial changes may contribute to the observed improvements in muscle mass and function, supporting the “gut-muscle axis” hypothesis.

## Discussion

This comprehensive study demonstrates that oral supplementation with *W. cibaria* Wikim0187, a probiotic strain isolated from kimchi, effectively attenuates age-related sarcopenia in aged mice through multiple coordinated mechanisms. The integrated findings provide strong evidence supporting the therapeutic potential of this probiotic strain for managing age-related muscle loss through a multifaceted approach that operates at molecular, cellular, tissue, and systemic levels.

Wikim0187 exerts cytoprotective effects against oxidative stress in muscle cells through its potential antioxidant properties. The antioxidant activity of LAB is attributed to peptidoglycan and teichoic acids in their cell walls that directly neutralize ROS, as well as LAB-derived metabolites such as exopolysaccharides with antioxidant properties [38]. This oxidative stress reduction is particularly important because skeletal muscle, with its high metabolic activity and abundant mitochondrial content, is especially susceptible to age-related accumulation of oxidative damage [12-16]. By reducing ROS-mediated signaling and FoxO activation, Wikim0187 may directly suppresses atrophy-related gene expression.

In this study, we found the substantial reduction in muscle atrophy–related genes MuRF-1 and Atrogin-1 across multiple hindlimb muscles (33–66% reduction), which encode E3 ubiquitin ligases central to ubiquitin–proteasome system–mediated protein degradation [15, 16, 39]. This suppression might be mediated through multiple signaling pathways, including FoxO and NF-κB, which are downregulated by Wikim0187 treatment. The increased Akt phosphorylation observed in the present study provides critical mechanistic insight, as Akt inhibits FoxO transcription factors through phosphorylation-mediated nuclear exclusion [40, 41]. This coordinated suppression of atrophy-related gene expression, together with preserved protein synthesis, represents an effective strategy for mitigating sarcopenia.

Parallel to the suppression of catabolic pathways, Wikim0187 treatment markedly increased the expression of myogenic regulatory factors MyoD and Myogenin, with particularly pronounced increases of 3.7-fold and 3.1-fold, respectively, in quadriceps muscle. The elevated Akt phosphorylation further activates the PI3K/Akt/mTOR pathway, a central regulator of protein synthesis through S6K and 4E-BP1 phosphorylation [42, 43]. This dual mechanism—simultaneous promotion of protein synthesis and inhibition of protein degradation—accounts for the observed increases in muscle mass (12–30%), grip strength (28%), and muscle fiber cross-sectional area (31–43%) over the 4-month treatment period. Notably, by comparing the functional performance of aged mice with the baseline data from healthy young mice (Supplementary Fig. S1), we confirmed that the W0187-treated group maintained grip strength at levels statistically comparable to the young baseline. This indicates that W0187 does not merely induce a transient improvement in aged subjects but effectively attenuates the age-related rate of functional decline, preserving a phenotype closer to a youthful, healthy state. This healthy baseline was established using a separate reference cohort of young mice (12 weeks old at the start of the study) that received oral administration for 4 weeks under identical experimental conditions, serving strictly to contextualize the magnitude of age-related functional decline and subsequent recovery.

In the present study, we found that administration of Wikim0187 modulates gut microbiota structure, which is significant correlated with markers of skeletal muscle integrity and function, supporting the gut–muscle axis hypothesis. Enrichment of SCFA–producing gut bacteria enhances intestinal barrier integrity and reduces systemic lipopolysaccharide translocation, thereby attenuating chronic low-grade inflammation [44]. This systemic modulation by microbiota-targeted interventions is crucial for maintaining metabolic homeostasis and mitigating muscle wasting, as supported by recent studies on the gut-muscle-immune axis [45]. SCFA–mediated activation of GPR43 and GPR109A further promotes immune tolerance and anti-inflammatory signaling [46]. To further investigate this immune regulation, we evaluated the serum levels of key systemic inflammatory cytokines (IL-12, TNF-α, IFN-γ, MCP-1, IL-10, and IL-6). Interestingly, the absolute circulating concentrations were at very low baseline levels across all aged mice, with no significant differences observed following *W. cibaria* Wikim0187 administration (Supplementary Fig. S3). This suggests that the protective effects of *W. cibaria* Wikim0187 against muscle loss are predominantly driven by localized metabolic and signaling modulations—such as the direct suppression of atrophy-related genes via gut-derived metabolites—rather than massive shifts in systemic circulating cytokines. Although we did not directly measure short-chain fatty acid (SCFA) levels, the finding that the relative abundance of the genera *Akkermansia* and *Bifidobacterium*, representative SCFA-producing gut microbes [47], was significantly increased in the gut by Wikim0187 administration further supports the gut–muscle axis hypothesis. Moreover, LAB-derived metabolites, including B vitamins, may contribute to immune regulation through effects on T cell differentiation and aryl hydrocarbon receptor signaling, enhancing immune tolerance [26, 27].

Indeed, our findings highlight the potential involvement of a broader gut-liver-muscle axis. Gut-derived SCFAs, produced by these enriched taxa, travel via the portal circulation to the liver to improve metabolic homeostasis and mitigate hepatic inflammation [48]. This optimized hepatic function can subsequently stimulate Akt-driven anabolic pathways in skeletal muscle through systemic mediators like IGF-1 [49]. At the molecular level, this creates a direct signaling blueprint connecting gut microbial shifts to muscle preservation: circulating SCFAs and systemic IGF-1 activate the PI3K/Akt/mTOR cascade in skeletal muscle. Activated Akt directly phosphorylates FoxO transcription factors, leading to their nuclear exclusion. Because FoxO is a primary transcriptional activator of muscle-specific E3 ubiquitin ligases, its removal from the nucleus effectively halts the transcription of target genes [39], directly explaining the substantial downregulation of MuRF-1 and Atrogin-1 observed in our study. Furthermore, the extensive effects of SCFAs have been well-documented to alleviate insulin resistance and systemic oxidative stress [50] and even extend to the central nervous system via the gut-brain axis [51]. Collectively, these multi-organ effects underscore how *W. cibaria* Wikim0187-induced SCFA production coordinates comprehensive metabolic benefits to preserve aging skeletal muscle.

Current therapeutic options for sarcopenia are limited to exercise and protein nutrition, which many elderly individuals cannot adequately access due to comorbidities, mobility limitations, or frailty [17]. The use of *W. cibaria* Wikim0187 as a therapeutic or preventive agent is particularly compelling. As a probiotic, Wikim0187 has “Generally Recognized as Safe” status and, unlike drugs with side effects, has the advantage of being easy to consume and having no side effects. Future clinical trials in human subjects are warranted to validate these preclinical findings, establish the efficacy of Wikim0187 supplementation in improving muscle mass, strength, and function in aging populations, and determine optimal dosing regimens and relevant patient populations for treatment.

Despite these promising findings, our study has several limitations that warrant consideration. First, regarding the *in vivo* model, the daily oral gavage induces physiological stress that could theoretically affect gut-systemic responses [52]. Although dietary administration via food or water is a more translationally relevant model, gavage was necessary to ensure the precise delivery of viable bacteria to aged mice. We controlled for this variable by administering an identical sham gavage to the vehicle group. Furthermore, based on our preliminary data (Supplementary Fig. S2), we selected the 1 × 10^9^ CFU dose because it demonstrated functional efficacy comparable to the 1 × 10^10^ CFU dose. Second, while we observed strong correlations between enriched bacterial taxa and improved muscle phenotypes, this is insufficient to establish definitive causality. Further studies employing fecal microbiota transplantation (FMT) into germ-free or antibiotic-depleted aged models are essential to conclusively verify whether the altered microbiota alone can recapitulate these protective effects. Finally, our *in vitro* C2C12 myoblast model, while successfully confirming fundamental cytoprotective properties, cannot fully capture the complex, multifactorial pathophysiology of *in vivo* sarcopenia, which involves systemic factors like inflammatory cytokines and motor neuron denervation [53].

## Conclusion

This study reveals that the kimchi-derived probiotic *Weissella cibaria* Wikim0187 exerts coordinated anti-sarcopenic effects by targeting both muscle protein turnover and the gut–muscle axis in aged mice. Wikim0187 is associated with the suppression of ubiquitin–proteasome–mediated protein degradation, enhanced Akt-driven anabolic signaling, and the upregulation of myogenic regulators, thereby improving muscle mass, fiber morphology, and strength rather than merely slowing decline. In parallel, Wikim0187 reshapes the gut microbiota, enriching taxa such as *Akkermansia* and *Bifidobacterium* that are positively associated with muscle integrity and negatively associated with atrophy-related markers, highlighting a microbiota-dependent mechanism for preserving skeletal muscle during aging. Future directions include randomized clinical trials in elderly populations to validate efficacy and safety, mechanistic studies combining gnotobiotic models with metabolomics to dissect host–microbe signaling, and combinatorial strategies pairing Wikim0187 with exercise or tailored nutritional interventions to test for additive or synergistic benefits on healthy muscle aging.

## Supplemental Materials

Supplementary data for this paper are available on-line only at http://jmb.or.kr.



## Figures and Tables

**Table 1:** Primer sequences for real-time PCR analysis