# Supplementary material for: Kimchi Probiotic Weissella cibaria Wikim0187 Attenuates Age-Related Muscle Loss: Association with Gut Microbiota Remodeling in Mice
Source: J Microbiol Biotechnol. 2026 Jul 17;36:e2607010. doi: 10.4014/jmb.2607.07010 (PMC13402211; doi:10.4014/jmb.2607.07010)
Supplement: Supplementary file 1 [file jmb-36-e2607010-supple.pdf]

J. Microbiol. Biotechnol. 2026. 36: e2607010

https://doi.org/10.4014/jmb.2607.07010

Supplementary Figures

Kimchi probiotic *Weissella cibaria* Wikim0187 attenuates age-related muscle loss: association with gut microbiota remodeling in mice

Sulhee Lee[1,3†], Ga Hee Choi[1,4†], Sang-Pil Choi[5], Namhee Kim[2], Young Seo Jang[1,6], Min-Sung Kwon[1], Young Joon Oh[1], Jeong Hyun Seo[7], Hwayeon Sun[7], Ji Ye Mok[7], Sang Min Park[7], Byungwook Lee[7], and Hak-Jong Choi[1*]

[1] *Kimchi Healthcare Research Group, World Institute of Kimchi, Gwangju 61755, Republic of Korea*

[2] *Kimchiome Bio-Resources Research Group, World Institute of Kimchi, Gwangju 61755, Republic of Korea*

[3] *Korean Culture Center of Microorganisms, Seoul 03641, Republic of Korea*

[4] *Division of Animal Science, Chonnam National University, Gwangju 61186, Republic of Korea*

[5] *Division of Radiation Biomedical Research, Korea Institute of Radiological and Medical Sciences, Seoul 01812, Republic of Korea*

[6] *Department of Biotechnology, Graduate School, Korea University, Seoul 02841, Republic of Korea*

[7] *Pharmsville Co., Ltd., Seoul 07793, Republic of Korea*

[†]These authors contributed equally to this work.

[*]Corresponding author

H.J. Choi, E-mail: hjchoi@wikim.re.kr

Supplementary Materials and Methods

Inflammatory Cytokine Levels

Blood samples were collected from experimental mice after euthanization, and serum was obtained by centrifugation at 3,000×*g* for 10 min. The levels of TNF-α, IL-12, IL-10, MCP-1, IL-6 and IFN-γ were determined by flow cytometry using a Cytometric Bead Array kit (BD Bioscience, San Jose, CA).

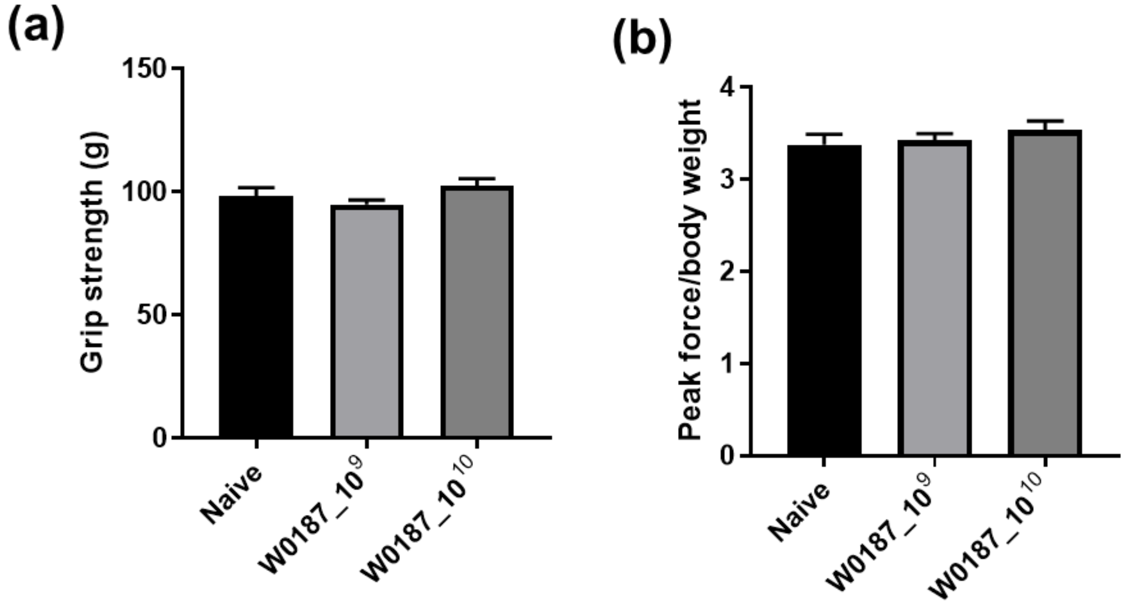

Fig. S1. Baseline muscle strength in young, healthy C57BL/6J mice. (a) Absolute forelimb grip strength (peak force) and **(b)**grip strength normalized to body weight. To establish a healthy functional baseline, 12-week-old young mice were acclimated and assessed under identical experimental conditions as the aged cohort. Data represent the 16-18 week age period following a 4-week administration of *W. cibaria* Wikim0187 (1×10^9 or 1×10^{10} CFU/mouse) or vehicle. There were no significant differences among the groups, indicating that the probiotic supplementation does not induce unnatural muscle hypertrophy or abnormal strength increases in already healthy young mice. Data are expressed as mean ± SEM

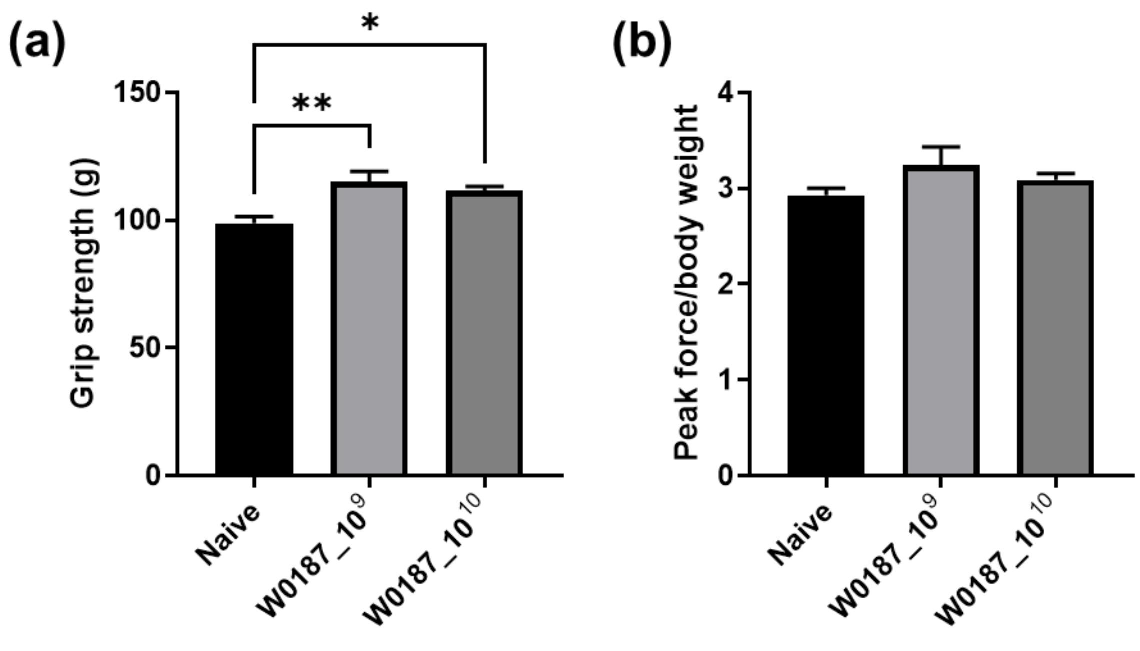

Fig. S2. Preliminary dose-response evaluation of *W. cibaria* Wikim0187 on muscle strength in aged mice. (a) Absolute forelimb grip strength and **(b)** grip strength normalized to body weight in aged mice treated with varying doses of *W. cibaria* Wikim0187 (1×10^9 CFU and 1×10^{10} CFU per mouse) or vehicle for 4 weeks. Both the 10^9 and 10^{10} CFU dosages significantly improved absolute muscle strength compared to the vehicle-treated group, with comparable efficacy. Based on these findings, physiological translatability, and the goal of minimizing potential chronic gavage stress, the 1×10^9 CFU dose was selected for the primary 4-month long-term intervention. Data are expressed as mean ± SEM (n=5 per group). Statistical analysis was performed using one-way ANOVA ($^{*}p<0.05$, $^{**}p<0.01$ vs. Naive).

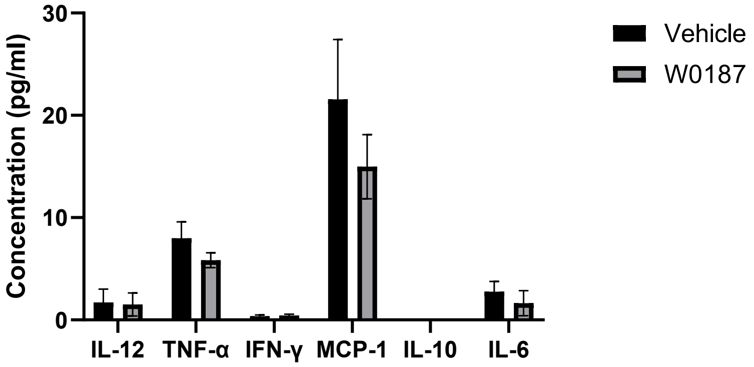

Fig. S3. Effects of *W. cibaria* Wikim0187 on systemic inflammatory cytokine levels in aged mice. Serum concentrations of interleukin-12 (IL-12), tumor necrosis factor-alpha (TNF-α), interferon-gamma (IFN-γ), monocyte chemoattractant protein-1 (MCP-1), interleukin-10 (IL-10), and interleukin-6 (IL-6) were quantitatively measured using a Cytometric Bead Array (CBA) assay. The absolute concentrations of these circulating cytokines were generally at low baseline levels across all mice. There were no statistically significant differences observed between the vehicle-treated and *W. cibaria* Wikim0187-treated groups. Data are expressed as mean ± SEM (n = 5 per group).
